# Platelet Storage Pool Deficiency and Elevated Inflammatory Biomarkers Are Prevalent in Postural Orthostatic Tachycardia Syndrome

**DOI:** 10.3390/cells11050774

**Published:** 2022-02-23

**Authors:** William T. Gunning, Paula M. Kramer, Jacob A. Cichocki, Beverly L. Karabin, Sadik A. Khuder, Blair P. Grubb

**Affiliations:** 1 Department of Pathology, University of Toledo, Toledo, OH 43614, USA; paula.kramer2@utoledo.edu (P.M.K.); jacob.cichocki@utoledo.edu (J.A.C.); 2 Department of Medicine, University of Toledo, Toledo, OH 43614, USA; beverly.karabin@utoledo.edu (B.L.K.); sadik.khuder@utoledo.edu (S.A.K.); blair.grubb@utoledo.edu (B.P.G.)

**Keywords:** POTS, tachycardia, cytokine, inflammation, syncope, platelet, storage pool deficiency, innate immune system activation

## Abstract

A significant number of postural orthostatic tachycardia syndrome (POTS) patients have platelet delta granule storage pool deficiency (δ-SPD). The etiology of POTS is unknown but a number of laboratories, including ours, have reported elevations of G-protein-coupled adrenergic receptor and muscarinic acetylcholine receptor autoantibodies in POTS patients, detected by a variety of techniques, suggesting that the disorder is an autoimmune condition. Thus, it could also be considered an inflammatory disease. In a pilot study, we investigated a limited number of platelet-related cytokines and chemokines and discovered many that were elevated. This case–control study validates our pilot study results that POTS patients have an activated innate immune system. Plasma of 35 POTS patients and 35 patients with unexplained bleeding symptoms and categorized as “non-POTS” subjects was analyzed by multiplex flow cytometry to quantify 16 different innate immune system cytokines and chemokines. Electron microscopy was used to quantify platelet dense granules. Ten of 16 biomarkers of inflammation were elevated in plasma from POTS patients compared to non-POTS subjects, with most of the differences extremely significant, with *p* values < 0.0001. Of particular interest were elevations of IL-1β and IL-18 and decreased or normal levels of type 1 interferons in POTS patients, suggesting that the etiology of POTS might be autoinflammatory. All POTS patients had δ-SPD. With a growing body of evidence that POTS is an autoimmune disease and having elevations of the innate immune system, our results suggest a potential T-cell-mediated autoimmunity in POTS characteristic of a mixed-pattern inflammatory disease similar to rheumatoid arthritis.

## 1. Introduction

Postural orthostatic tachycardia syndrome (POTS) is a condition of orthostatic intolerance with a racing heart and a multitude of symptoms, induced upon standing and relieved when becoming supine [1]. Common nonspecific symptoms reported with POTS include exercise intolerance, fatigue, lightheadedness, palpitations, nausea, headache, diminished concentration (“brain fog”), near syncope, and syncope. It is a debilitating disorder, affecting an estimated 1% of the population and likely more with the numerous reports in the literature of COVID-19 “long haulers” exhibiting many of the symptoms described by POTS patients; a number of these patients have actually been diagnosed with POTS [2,3,4,5,6]. The disorder was likely first described in 1871 by Da Costa, who described all of the features of POTS mentioned above; it was formally monikered in 1993 by Schondorf and Low, yet, even today, it is not well recognized in the medical community, nor has an etiology been established [1,7]. Unfortunately, as many as 75% of affected individuals are misdiagnosed or referred for psychological consultation [8,9,10,11]. The diagnosis requires the presence of chronic orthostatic intolerance associated with an increased heart rate of ≥30 beats per minute (BPM) from the supine or sitting basal rate, or a rate that exceeds 120 BPM when standing or by an upright tilt test that occurs within 10 min [1,12]. There is also an absence of orthostatic hypotension, a duration of symptoms for at least 6 months, and the absence of a number of conditions that could explain sinus tachycardia [1]. There is a growing body of evidence suggesting that POTS is an autoimmune disease [13,14,15,16,17,18].

We have reported that a number of symptoms observed in POTS may be related to a platelet deficiency of granules that contain stores of serotonin, called platelet delta granule storage pool deficiency (δ-SPD) [8]. Platelet δ-SPD is an autosomal dominant inherited disorder but may also be acquired; there are many documented genetic associations including autoimmune diseases [9,10]. We have empirical evidence that the acquired δ-SPD may be related to viral infection and/or chronic inflammation and, based upon this, we explored a number of cytokines and chemokines related to platelet activation in a limited number of POTS patients that had elevations of G-protein-linked autoantibodies against the alpha 1 adrenergic receptor [11]. We reported that a number of inflammatory biomarkers were significantly elevated, but, as a pilot study, our experimental design was incomplete. The platelet is an essential element of the innate immune system, especially in defense of viral infections [12,13,14]. With associations of viral infections as a potential etiology of POTS and evidence that many COVID-19 long haulers are developing POTS, platelets may also play a significant role in the etiology or perpetuation of the disorder.

The purpose of the current study was to expand the number of inflammatory biomarkers we had previously evaluated in a case–control study of POTS and non-POTS patients. Our hypothesis was that POTS has elevations of cytokines and chemokines indicating innate immune cell activation characteristic of an autoinflammatory disease. These biochemicals may be induced by the release of substances contained in the platelet storage pool that prolong autoimmunity. The specific aims of the investigation were (1) to determine the potential that POTS might be an autoinflammatory condition rather than an autoimmune disease, and (2) to validate our previous results that POTS patients have significant elevations of innate immune system biochemicals that are related to the platelet as an immune cell.

## 2. Materials and Methods

### 2.1. Patients

Our retrospective case–control study was approved by the Institutional Review Board of The University of Toledo Medical Center. Platelet-poor plasma (PPP) from 35 patients diagnosed with POTS and 35 patients without POTS was assayed for a number of biomarkers of inflammation that we have previously reported [11]. The specific inflammatory biomarkers included in this investigation were selected based upon descriptions in the literature related to platelet activation [15,16,17,18,19,20]. All POTS patients had histories of orthostatic intolerance manifested by orthostatic tachycardia, weakness, light-headedness, fatigue, and near syncope for at least 6 months or longer and were diagnosed with primary POTS in our Syncope and Autonomic Disorders Clinic. The diagnosis was based upon clinical history, physical examination, and head-upright tilt table analysis in the fasting state. Blood chemistry analysis and thyroid profile analysis were included during diagnostic workups. These patients had also exhibited bleeding symptoms and peripheral blood had been submitted to assess platelet dense granules for δ-SPD to explain these symptoms. The control subjects did not include anyone diagnosed with POTS, but they had been evaluated for a potential platelet function disorder and found to have a normal number of platelet dense granules.

All subjects had a complete blood cell count (CBC) and a mean number of platelet dense granules determined. We have previously reported that more than 80% of patients diagnosed with POTS have δ-SPD.

### 2.2. Platelet Preparations for Electron Microscopy

**Figure 1:**
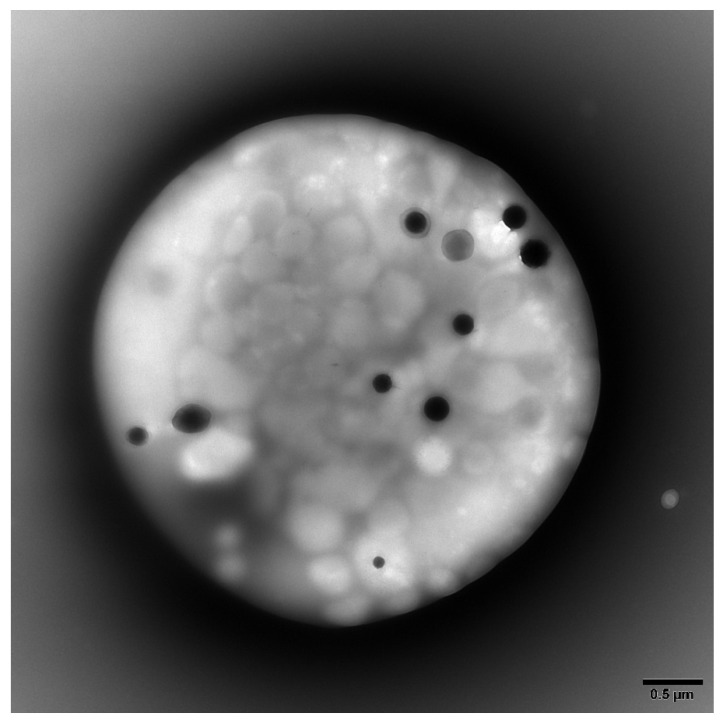
Representative transmission electron microscopy image of a whole mounted and air-dried platelet. Dense granules appear as opaque round bodies (normal = 4–6/platelet; range 0–30/platelet), whereas the ill-defined gray bodies are alpha granules (50–80/platelet).

Platelet-rich plasma (PRP) was obtained from whole blood by centrifugation at room temperature for 15 min at 200 g. Electron microscopy coated copper grids used for platelet support were washed with deionized water following PRP incubation and air-dried. A FEI Tecnai G2 Spirit BioTwin transmission electron microscope (TEM, Hillsboro, OR) was used to determine the average number of DG/PL (Figure 1). Previous studies from this laboratory have established a normal value of 4.64 ± 0.11 (mean ± 1 SE DG/PL), consistent with the established literature [9,21,22].

### 2.3. Inflammation Biomarker Preparations

A custom RayPlex^®^ Human Multiplex Bead Array was purchased to assess 16 cytokine/chemokine biomarkers from RayBiotech, Inc. (Peachtree Corners, GA 30092, USA), including IL-1β, IL-6, IL-8, IL-10, IL-17, IL-18, IL-21, INFα, INFβ, INFγ, TNFα, CD30, CD40, sCD40L (CD154), MCP-1 (chemokine ligand 2/CCL2), and CCL5 (RANTES), all of which have been associated with inflammation and/or platelet activation. The multiplex bead system for flow cytometry allowed for simultaneous quantification of the cytokines/chemokines. During validation of the custom beads, it was determined that INFα could not be included due to detection limits, and a standard sandwich-based ELISA was utilized to quantitate the interferon. Validation and quantitation for all targets was made by comparison with specific protein concentrations for each cytokine/chemokine from standard curves. All samples were analyzed by RayBiotech in Peachtree Corners, Georgia.

### 2.4. Statistical Methods

Unless otherwise stated, data are presented as mean + 1 standard error of the mean (SE). Descriptive statistics were calculated using R statistical software (R Core Team, R Foundation for Statistical Computing, Vienna, Austria. URL https://www.R-project.org/, accessed on 2 August 2021). Univariate analysis of variance, Tukey HSD, and linear discriminant analysis were utilized to analyze cytokine and chemokine concentration variances between groups, and a Student t test and Mann–Whitney Rank Sum test were used to compare the mean PL/DG and CBC results between groups. SigmaPlot software (version 14.5, Systat Software, Inc. Palo Alto, CA, USA) was also used to produce graphs for the manuscript.

## 3. Results

The mean age of subjects in study groups was not statistically different (mean age for POTS patients was 22.2 ± 2.9 and non-POTS subjects was 22.0 ± 2.0). However, POTS patients were found to have a mean of 2.65 ± 0.22 DG/PL (normal = 4.64 ± 0.11), which is consistent with δ-SPD, in contrast to non-POTS subjects, who had normal numbers of DGs (4.95 ± 0.11 DG/PL) (*p* < 0.0001). There was no statistical correlation found with any of the hematologic factors measured (CBCs), except the hematocrit between groups. The POTS patients had a lower hematocrit (33.1%; lower than normal (36.1–44.3%)) compared with non-POTS subjects (44.1%, *p* < 0.008).

Highly significant differences were found between groups for 11/16 of the cytokine/chemokine plasma concentrations evaluated (Table 1). Differences in IL-1β, IL-10, IL-17, INFϒ, and RANTES (CCL5) were striking, with each having *p* values < 0.0001. All differences identified were increased concentrations of innate immune cytokines in POTS subjects, except for the type 1 interferon INFα, which was decreased in POTS (0.06 ± 0.04) when compared to the plasma concentration of the control group (223 ± 67, *p* < 0.002). No difference was found between groups for CD40, CD40L (CD154), IL-8, INFβ, or TNFα.

## 4. Discussion

This case–control study was intended to validate a pilot study of inflammatory biomarkers, presumably related to platelets, obtained from POTS patients having elevated G-protein-coupled autoantibodies [11]. Platelets play a crucial role in hemostasis but also have a significant role in both innate and adaptive immunity [16,23,24]. Platelets contain cytokines and chemokines known to modulate the effects of leukocytes as either pro- or anti- inflammatory agents [16,25,26].

Our pilot study provided data that suggested that the innate immune system might have a significant role in the etiology of POTS. In fact, our limited data suggested that POTS could be T-cell-mediated. Unfortunately, we did not include an investigation of type 1 interferons to allow us to distinguish an autoinflammatory process from autoimmunity. In addition, we did not evaluate IL-17 levels in plasma; the cytokine plays a significant role in both innate and adaptive immune responses and there is a growing body of evidence suggesting that IL-17 is elevated in autoimmune disorders [27,28].

Specific aim 1 addressed the potential that POTS might be an autoinflammatory condition rather than an autoimmune disease. To evaluate this, we measured the plasma concentrations of IL-1β, IL-18 (an IL-1 “family” member), the type 1 interferons INFα and INFβ, and the type 2 interferon INFϒ. IL-1 and type I interferons are diametrically opposed; inflammatory disorders with elevations of IL-1 are categorized as autoinflammatory whereas conditions with elevations of type 1 interferons are characteristic of autoimmune diseases [29,30]. POTS patients evaluated in this study had significant elevations of IL-1β compared to non-POTS subjects (*p* < 0.0001), as well as elevations of IL-18 (*p* < 0.009). Interferon alpha was significantly decreased in POTS compared to non-POTS subjects (*p* < 0.002), whereas no significant difference was found for plasma levels of INFβ. Elevations of type 1 INF appear to be critical mediators of autoimmune disease [31]. These data provide evidence that POTS is an autoinflammatory condition. However, we and others have reported that POTS appears to be an autoimmune disorder based upon the identification of a variety of autoantibodies detected in the blood of POTS patients [32,33,34,35,36]. Antibody production is characteristic of an activated adaptive immune system or that an individual has been exposed to a foreign antigen; elevations of autoantibodies (antibodies against self) are a hallmark of autoimmune disease.

Our data strongly suggest that POTS is an autoinflammatory condition, yet the current literature suggests it is an autoimmune disease. Pure autoinflammatory diseases are strongly associated with fever; POTS patients are known to have difficulty regulating body temperature. These conflicting hypotheses can be rationalized by considering that POTS has a “mixed” inflammatory signature. There are a number of recent reports in the literature providing evidence of mixed-pattern diseases [29,37,38,39]. We found that POTS patients had elevations of the proinflammatory cytokines IL-1β, IL-6, IL-18, and INFϒ, which have been reported in both autoinflammatory and autoimmune diseases and, potentially, are a result of abnormal NK cell function [40].

Other elevated proinflammatory biomarkers included IL-17 (*p* < 0.0001), MCP-1 (*p* < 0.0002), and RANTES (*p* < 0.0001; an indicator of platelet activation), whereas the proinflammatory TNFα was found to be at a similar concentration to that in non-POTS subjects. Interestingly, our pilot study found elevations of TNFα [11]. Tumor necrosis factor alpha is a cytokine of the innate immune system involved in acute phase reactions and produced primarily by activated macrophages and by T helper and NKT cells in response to IL-1 [41]. More recently, TNFα has been reported to induce the inflammasome-independent production of IL-1β, causing autoimmunity [42]. CD30, which is part of the TNF family and regulates cell proliferation, was elevated in both our pilot study and this investigation. 

CD30 is not expressed on resting or naive T and B cells but is a biomarker of both types of activated lymphocytes. It is elevated in both autoimmune and chronic inflammatory diseases [43]. It is released from the surfaces of activated T cells, B cells, and NK cells and has been used as a biomarker in a number of studies to predict renal allograft rejection [44]. CD30 is cleaved from the surfaces of these activated cells and circulates in the peripheral blood as soluble (s)CD30. Elevation of CD30, among other biomarkers, has been reported to be associated with an increased risk for the development of non-Hodgkin lymphoma, as well as resulting from viral infection such as EBV and HIV [45]. We have postulated that a viral infection may be the initiating stimulus for the development of autoantibodies in POTS; the results reported here are consistent with the premise but it requires additional research [9,11].

Other regulatory cytokines found to be elevated in this study were IL-10 (*p* < 0.0001) and IL-21 (*p* < 0.003); these biomarkers would be expected to be elevated as feedbacks to turn-off the innate immune system. One of the major functions of IL-10 is a suppressive effect on T cell subsets as an anti-inflammatory cytokine, principally secreted by Treg cells [41]. IL-21 is produced by CD4 + T cells, natural killer T cells (NKT), and follicular helper T cells and induces B cell proliferation and differentiation into plasma cells [41]. Of particular interest, elevation of IL-21 has been reported in autoimmune diseases including celiac disease (CD), rheumatoid arthritis (RA), and systemic lupus erythematous (SLE) [46,47,48].

Cytokines and chemokines that we found elevated in POTS patients, in contrast to our non-POTS group, and that have been related to autoimmune diseases, include not only IL-21, but also IL-1β, INFϒ, CD30, and IL-17 [49,50,51]. Although these biomarkers are elevated in autoimmunity, as stated previously, they also play significant roles in both innate and adaptive immune responses. Our data support a mixed-pattern inflammatory disease to best categorize POTS [29].

The second specific aim of our study was to validate previous results that POTS patients have significant elevations of innate immune system biochemicals that are related to the platelet as an immune cell. We have validated our previous results, but what about the platelet and its role in POTS? POTS patients were found to be platelet delta granule storage pool deficient (δ-SPD), with a mean of 2.65 ± 0.22 DG/PL, compared to our non-POTS group, which had normal numbers of DGs (4.95 ± 0.11 DG/PL) (*p* < 0.0001; normal = 4.64 ± 0.11). As previously mentioned, δ-SPD is usually considered an autosomal dominant inherited disorder but may also be recessive and/or acquired. Consequently, why do POTS patients have platelet δ-SPD? If the subjects in our study had inherited δ-SPD, it might be possible that δ-SPD is a risk factor for susceptibility to POTS. However, it may be a biomarker of innate immune system activation. We have empirical data that suggest that platelet δ-SPD may be acquired in viral infections (Epstein–Barr virus) and in cases of chronic inflammation. Platelet δ-SPD has been previously described in autoimmune diseases including SLE, RA, and Sjögren’s syndrome [52,53]. Since platelets contain cytokines and chemokines common in both innate and adaptive immunity, it is possible that the association we have found with platelet δ-SPD is evidence that this comorbidity is an acquired disorder, suggesting that the platelet may drive the mixed inflammatory profile of POTS [16,20,54,55]. This is an unanswered question that needs to be addressed in future investigations, requiring assessments of POTS patients who have recovered from the disorder. Ideally, such an investigation would include platelet analysis at the time of POTS diagnosis, and reassessed subsequently, at a sufficient time interval after the disorder’s resolution.

There are a number of limitations of our study. The study was initiated at the height of the COVID-19 pandemic, when all existing prospective investigations at our institution were put on hold and new prospective protocols were not considered by our IRB office. Thus, we were relegated to a retrospective study with all of the inherent problems related to such investigations. We lacked specific clinical histories for samples including Beighton scores used to assess hyperflexibility; some of the POTS patient group may have had hypermobility spectrum disorders (HSD) or hypermobile Ehlers–Danlos syndrome (h-EDS), which can be associated with easy bruising. The potential that δ-SPD may be associated with HSD or EDS rather than POTS cannot be ruled out. We identified POTS samples based upon clinical test orders of one of our authors (BPG). Non-POTS samples were selected based upon identifying tests that had been ordered by a number of hematologists interested in the diagnosis of unexplained bleeding; the selected samples had normal test results. Our control group should not be considered “normal subjects” as samples in this group had been evaluated for platelet δ-SPD in patients with unexplained bleeding symptoms. It is entirely possible that a few of the non-POTS samples might have had an under-lying autoimmune disease. Regardless, most of the *p* values generated by the linear discriminant analysis utilized to analyze cytokine and chemokine concentration variances between groups were extremely significant, with *p* values < 0.0001. We have initiated a large prospective study to assess an expanded list of cytokines/chemokines and to ensure that our healthy control group does not include subjects with bleeding symptoms or other medical conditions. This study is utilizing questionnaires to objectively score bleeding history, hyperflexibility, and dysautonomia (COMPASS 31).

## 5. Conclusions

In conclusion, we have validated results described in a previous report that POTS patients have elevated biomarkers of an activated innate immune system [11]. Although we postulated that POTS is an autoimmune disease mediated by T cells, similar to RA, psoriasis, systemic sclerosis, multiple sclerosis, and type-1 diabetes [42,51,56,57], the data provided herein suggest that POTS is a mixed inflammatory pattern disease. The elevated IL-1 family cytokines IL-1β and IL-18 are significantly elevated in POTS, a characteristic of an autoinflammatory disease. However, we and others, using a variety of techniques, have reported a number of different autoantibodies in POTS patients that would be consistent with an autoimmune disorder. Type 1 interferons that are characteristic of autoimmune disease were not elevated in our POTS patients. We currently have a prospective study in progress to examine the hypothesis that POTS is a T-cell-mediated disorder similar to RA.

## Figures and Tables

**Table 1:** Inflammatory biomarkers in POTS patients.

Cytokine/ Chemokine	POTS (*n* = 35) (pg/mL)	Non-POTS (*n* = 35) (pg/mL)	*p* Value	Source	Major Function
CD30	3638 ± 822	160 ± 12	*p* < 0.0002	Activated T and B	Regulates cell proliferation
CD40	340 ± 165	452 ± 171	ns	B cell, Mac	TLR7 PLT–neutrophil tethering
CD40 L (CD154)	31 ± 13	6.7 ± 0.8	ns	Platelets, Mono	Recruits neutrophils and monocytes
IL 1β	38 ± 8	4.4 ± 0.9	*p <* 0.0001	Mono/Mac, PLTs	Proinflammatory
IL-6	119 ± 18	58 ± 9	*p* < 0.003	Th Cells, Mac	Differentiates B cells to plasma cells
IL-8 (CXCL8)	145 ± 49	157 ± 25	ns	Mono, Neutro	Chemotaxis, proinflammatory
IL 10	24 ± 4	5.5 ± 1.0	*p* < 0.0001	T cell	Anti-inflammatory
IL-17	93 ± 20	4.2 ± 0.7	*p* < 0.0001	Th17	Proinflammatory
IL-18	207 ± 67	21 ± 9	*p* < 0.009	Mono	Proinflammatory, IL-1 family
IL 21	9025 ± 1875	2937 ± 517	*p* < 0.003	T cell	Controls NK and T cells
INFα	0.06 ± 0.04	223 ± 67	*p* < 0.002	Leukocytes	Anti-viral, phagocyte cell activation
INFβ	8219 ± 2230	6334 ± 3267	ns	Fibroblasts	Anti-viral, anti-proliferative
INFγ	8.5 ± 1.7	1.2 ± 0.2	*p <* 0.0001	NK, Th_1_	Antiviral, increases Neut and Mono function
MCP1 (CCL2)	441 ± 102	13 ± 2	*p* < 0.0002	Endo, PLT	Recruits monocytes
RANTES (CCL5)	13706 ± 3022	517 ± 297	*p* < 0.0001	Platelet, NK, T	Chemotactic for T cells
TNFα	972 ± 250	506 ± 120	ns	Mono, NK	Proinflammatory

Elevations of cytokines/chemokines are in red font. Decreases in cytokines/chemokines in blue font.

## Data Availability

All data that were generated during this research project are included in the manuscript.
